# Interaction of Flavonoids from *Woodwardia unigemmata* with Bovine Serum Albumin (BSA): Application of Spectroscopic Techniques and Molecular Modeling Methods

**DOI:** 10.3390/molecules22081317

**Published:** 2017-08-09

**Authors:** Rui Ma, Hong Pan, Tao Shen, Peng Li, Yanan Chen, Zhenyu Li, Xiaxia Di, Shuqi Wang

**Affiliations:** 1 School of Pharmaceutical Sciences, Shandong University, Jinan 250012, China; ma_rui_shandong@163.com (R.M.); panhong0011@yeah.net (H.P.); shentao@sdu.edu.cn (T.S.); 2 Shandong Institute of Environmental Science, Jinan, 250012, China; pengli_hjxy@126.com; 3 Jinan Institute of Measurement and Testing, Jinan, 250002, China; ych0845tg@163.com; 4 Department of Pharmacy, Shandong Provincial Hospital Affiliated to Shandong University, Jinan, 250021, China; lizhenyusdu@126.com; 5 Faculty of Pharmaceutical Sciences, University of Iceland, Reykjavik, 107, Iceland; dixiaxia_ic@sina.com

**Keywords:** *Woodwardia unigemmata*, multidrug resistance, doxorubicin-resistant K562/A02 cells, bovine serum albumin, molecular docking

## Abstract

Phytochemical investigation on the methanol extract of *Woodwardia unigemmata* resulted in the isolation of seven flavonoids, including one new flavonol acylglycoside (**1**). The structures of these compounds were elucidated on the basis of extensive spectroscopic analysis and comparison of literature data. The multidrug resistance (MDR) reversing activity was evaluated for the isolated compounds using doxorubicin-resistant K562/A02 cells model. Compound **6** showed comparable MDR reversing effect to verapamil. Furthermore, the interaction between compounds and bovine serum albumin (BSA) was investigated by spectroscopic methods, including steady-state fluorescence, synchronous fluorescence, circular dichroism (CD) spectroscopies, and molecular docking approach. The experimental results indicated that the seven flavonoids bind to BSA by static quenching mechanisms. The negative ΔH and ΔS values indicated that van der Waals interactions and hydrogen bonds contributed in the binding of compounds **2**–**6** to BSA. In the case of compounds **1** and **7** systems, the hydrophobic interactions play a major role. The binding of compounds to BSA causes slight changes in the secondary structure of BSA. There are two binding sites of compound **6** on BSA and site I is the main site according to the molecular docking studies and the site marker competitive binding assay.

## 1. Introduction

Multidrug resistance (MDR) renders the insensitivity of cancer cells to a diverse panel of anticancer agents, and it is the largest obstacle to cancer chemotherapy. A wide variety of natural products belonging to different classes have been found to have MDR-modulating activity [1,2]. Nevertheless, none of the MDR-reversal agents currently is available for clinical use, as they suffered from unpredictable pharmacokinetic behavior and toxicity [3]. Therefore, the exploration and development of potent and safe MDR reversal agents is still urgently needed.

Serum albumin (SA), a type of globular protein, is the most plentiful blood protein in mammals. The distribution of drugs is usually directed by SA, as most drugs circulate in plasma and reach the targeted tissues via binding to SA [4]. Therefore, the binding of drugs to SA turned into an essential determinant of pharmacokinetics restricting the unbound concentration and affecting distribution and elimination. Bovine serum albumin (BSA) is an important mode protein, which contains 583 amino acids with twenty tyrosyl (Tyr) residues and two tryptophan (Trp134, Trp213) residues [5]. Its structure looks like heart-shaped and that it is composed of three specific homologous domains I, II and III. Each domain has A and B subdomains. The principal regions of drugs’ binding sites of albumin are often located in hydrophobic cavities in sub-domains IIA and IIIA. Site I (located in subdomain IIA) is a selective binding site for heterocyclic anions, while site II (located in subdomain IIIA) is selected by aromatic carboxylates. Recently, BSA is frequently used as a model protein for the evaluation of interaction between drugs and protein because of its structural similarity (75.6%) with human serum albumin (HSA), low cost, ready availability and some usual ligand binding properties [6].

Ferns as a very ancient family of plants can predate the beginning of the Mesozoic era, 252 million years ago. Various types of natural compounds have been discovered from different fern species during the last decade [7,8]. In the current work, an extract of *Woodwardia unigemmata* (Makino) Nakai was chemically investigated and the obtained compounds were tested with MDR reversing effects. 

In our ongoing research for plant-derived MDR-reversal agents [9,10], we report the isolation and structure determination of seven flavonoids, including one new compound, from the whole plants of *W. unigemmata.* Compounds **1**–**7** were evaluated for their cytotoxicity towards K562 cells and multidrug resistance reversing activity on human myelogenous leukemia cells. Furthermore, compounds binding to BSA are studied using multi-spectroscopic and molecular modeling methods. Interaction information from quenching mechanisms, binding parameters, thermodynamic parameters and binding modes is reported in the present work. Molecular interactions were characterized using molecular docking studies.

## 2. Results and Discussion

### 2.1. Structure Elucidation of Compounds ***1**–**7***

**Figure 1:**
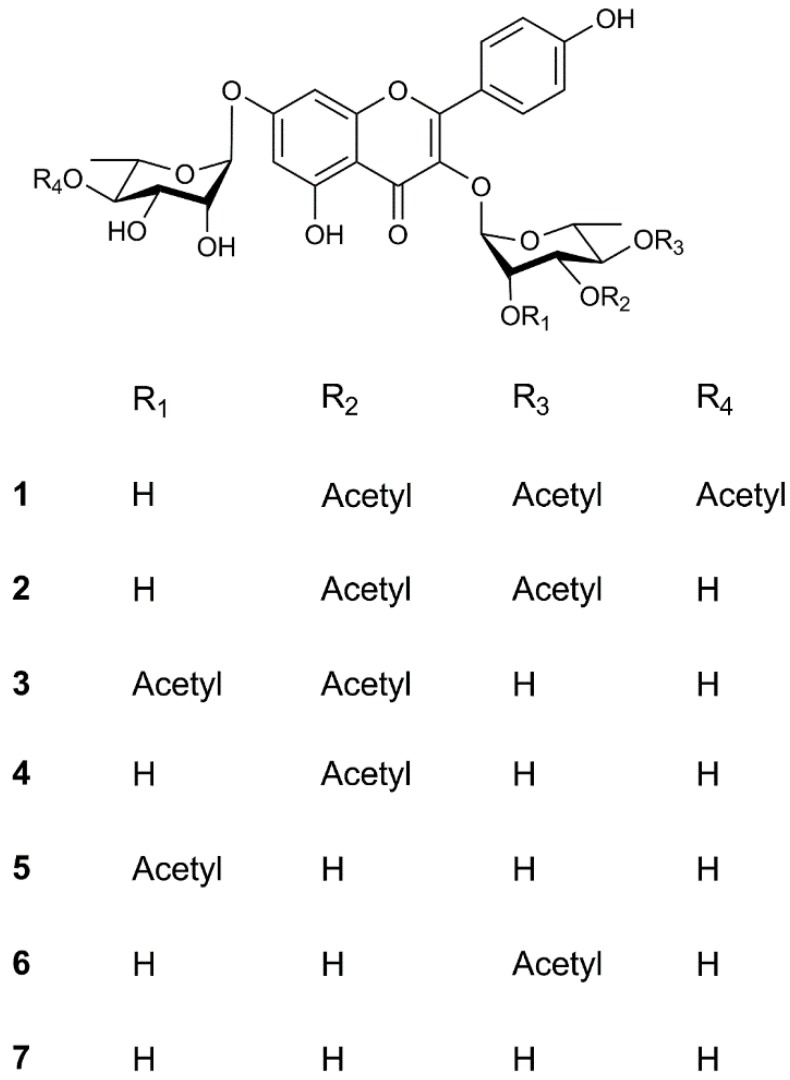
Structures of compounds **1**–**7**.

The methanol extract of the rhizomes of *W. japonica* was fractionated and purified by repeated column chromatography as described in the experimental section, leading to the isolation of a new compound **1** along with six known compounds **2**–**7** (Figure 1). 

Compound **1** was obtained as an amorphous powder with the molecular formula C_33_H_36_O_17_, as determined by HR-ESI-MS (*m*/*z* 705.2027 [M + H]^+^; calc. 705.2025). The ^1^H- and ^13^C-NMR (Table 1) spectra indicated that **1** was a flavonol with glycosidic and acyl moieties. The chemical shift and coupling constant data of the aromatic protons together with their corresponding ^13^C-NMR chemical shifts obtained from Heteronuclear Single Quantum Correlation (HSQC) and Heteronuclear Multiple Bond Correlation (HMBC) experiments confirmed the identity of kaempferol as the aglycone [11]. The ^1^H-NMR also indicated the presence of two sugar moieties with two anomeric protons at δH 5.64(^1^H, brs, H-1′′) and 5.58 (^1^H, brs, H-1′′′), which correspond to the carbon signals at δC 100.5 and 98.1 in the HSQC spectrum. Thus, the glycosyl moiety of 1 consisted of two sugar units. Two rhamnosyl moieties were presumed by analysis of the ^13^C-NMR data for monosaccharide. After sugar composition analysis, the presence of two l-rhamnose was confirmed [12,13]. The α-configuration of the rhamnose units were observed from the slight broadening of the appropriate H-1′′ and H-1′′′ signals [14]. The presence of the HMBC correlations between the rhamnosyl anomericproton H-1′′ at δH 5.64 and the resonance of C-3 at δC 134.1, and between the rhamnosyl anomericproton H-1′′′ at δH 5.58 and the resonance of C-7 at δC 130.6 suggested glycosidation at C-3 and C-7.

**Figure 2:**
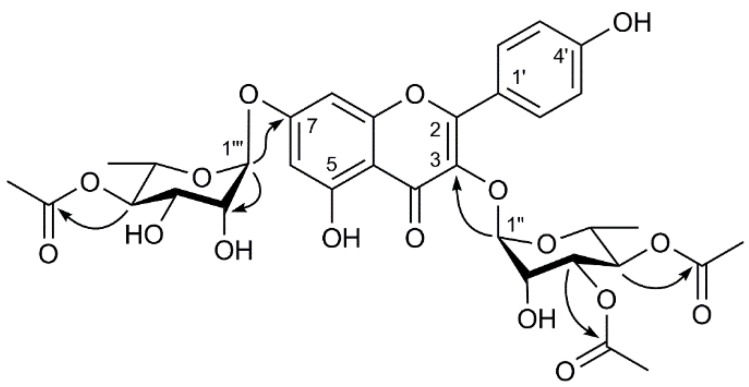
Key HMBC correlations of compound **1**.

The remaining sub-structure of **1** was deduced by analysis of the ^1^H-, ^13^C- and 2D-NMR spectroscopic data to possess three acetoxy groups [δH 2.13 (3H, s), 2.05 (3H, s) and 1.99 (3H, s)]. The acetyl groups were determined to be linked at the C-3′′, C-4′′ and C-4′′′ due to the presence of the cross-peaks between in the HMBC spectrum (Figure 2). Accordingly, the structure of **1** was elucidated as kaempferol3-*O*-α-l-(3,4-*O*-di-acetyl) rhamopyranoside-7-*O*-α-l-(4-*O*-acetyl) rhamnopyranoside.

The six known compounds crassirhizomoside B (**2**), crassirhizomoside C (**3**), kaempferol 3-*O*-α-l-(3-*O*-acetyl) rhamopyranoside-7-*O*-α-l-rhamnopyranoside (**4**), kaempferol 3-*O*-α-l-(2-*O*-acetyl) rhamopyranoside-7-*O*-α-l-rhamnopyranoside (**5**), sutchuenoside A (**6**), and kaempferol 3-*O*-α-l-rhamopyranoside-7-*O*-α-l-rhamnopyranoside (**7**) were identified on the basis of their spectroscopic profiles and comparison to the published data [15,16,17].

### 2.2. Pharmacological Studies

To determine the reversal effect of compounds **1**–**7** on resistant tumor cells, the cytotoxicity of the compounds towards K562, and their MDR variants K562/A02 cells was first measured by the MTT method. All compounds showed no cytotoxicity to both cell lines. The ability of compounds **1**–**7** to reverse MDR to doxorubicin (DOX) in cancer cells was investigated at a non-cytotoxic concentration (10 μM). Those compounds overcame the multidrug resistance (MDR) for doxorubicin-resistant K562/A02 cells, and the tested compounds showed the remarkable reversal effect (Table 2). Verapamil was a positive control. Those compounds are potential natural products that deserve more investigations to develop novel drugs against multifactorial drug-resistant cancers.

### 2.3. Fluorescence Quenching of BSA Induced by Compounds

Fluorescence measurements were performed to investigate whether compounds **1**–**7** interact with BSA. The intrinsic fluorescence of BSA is caused by Trp, Tyr, and Phe residues. Trp residues dominate the fluorescence spectrum of BSA due to its high quantum yield and the ability to quench the emission of Tyr and Phe residues through energy transfer [6]. BSA intramolecular forces and conformation can be affected and changed by small molecule binding.

To correct the inner filter effects of compounds and BSA, fluorescence intensity was calculated as:

(1) $$F_{\mathrm{cor}}=F_{\mathrm{obs}}10\frac{1}{2}\left(A_{\mathrm{ex}}+A_{\mathrm{em}}\right),$$

where F_cor_ and F_obs_ are the corrected and observed fluorescence intensities, respectively, and A_ex_ and A_em_ are the absorption of the systems at the excitation and emission wavelength, respectively. All fluorescence data mentioned in this work is intensity corrected.

**Figure 3:**
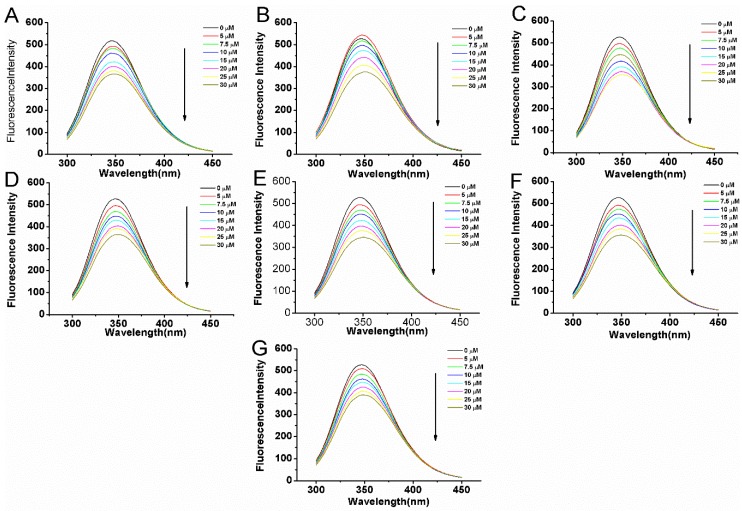
The fluorescence quenching spectra of BSA (5 μM) with variable concentrations of compounds **1** (**A**), **2** (**B**), **3** (**C**), **4** (**D**), **5** (**E**), **6** (**F**), **7** (**G**) at the excitation wavelength (280 nm) in 0.05 mol L^−1^ Tris-HCl, pH 7.4. The concentrations of compounds varied from 0 to 30 μM.

The fluorescence quenching spectra of BSA at various concentrations of compounds are shown in Figure 3. The Figure 3A–G indicates the compounds’ concentration dependent fluorescence quenching of BSA in the presence of 0–30 µM compounds. Despite the considerable fluorescence quenching in BSA, the emission maxima remained unchanged throughout the addition.

**Figure 4:**
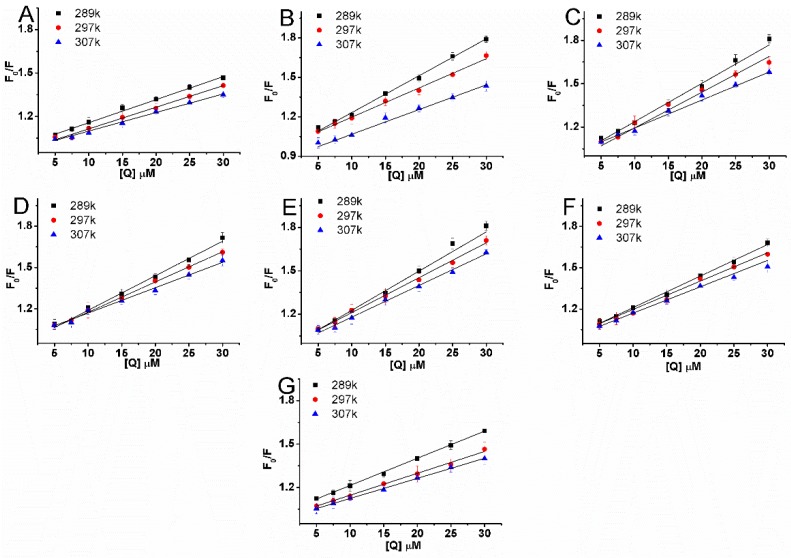
Stern–Volmer plots for BSA (5 μM) quenched by compounds **1** (**A**), **2** (**B**), **3** (**C**), **4** (**D**), **5** (**E**), **6** (**F**), and **7** (**G**) at different temperatures in 0.05 mol L^−1^ Tris-HCl, pH 7.4. The concentrations of compounds varied from 0 to 30 μM.

Fluorescence quenching is characterized by two types of mechanisms, usually classified as dynamic and static. Dynamic quenching refers to a process that involves the fluorophore and the quencher coming into contact during transient existence of the excited state, whereas static quenching refers to the formation of fluorophore-quencher complex [18]. Fluorescence quenching is described by the Stern–Volmer equation:

(2) $$\frac{F_{0}}{F}=1+K_{\mathrm{SV}}\left[Q\right]=1+K_{q}t_{0}\left[Q\right],$$

where F_0_ and F are the fluorescence intensities before and after the addition of the quencher, respectively, k_q_ is the bimolecular quenching constant, τ_0_ is the lifetime of the fluorophore in the absence of the quencher (τ_0_ = 10^−8^ s), [Q] is the concentration of the quencher, and K_sv_ is the Stern–Volmer quenching constant. Hence, the Stern–Volmer equation was applied to determine K_sv_ and k_q_ by linear regression of F_0_/F_sv_ [Q] [19]. K_sv_ is shown in Table 3. Figure 4 shows the modified Stern–Volmer plots of the fluorescence quenching of BSA by compounds at different temperatures (289 K, 297 K and 307 K).

**Figure 5:**
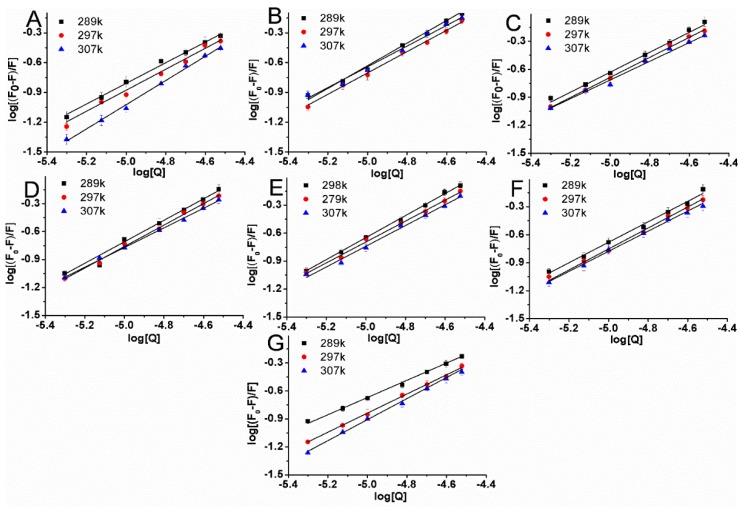
Plots of Log [(F_0_ − F)/F] vs. Log [Q] for BSA (5 μM) quenched by compounds **1** (**A**), **2** (**B**), **3** (**C**), **4** (**D**), **5** (**E**), **6** (**F**), and **7** (**G**) at different temperatures in 0.05 mol L^−1^ Tris-HCl, pH 7.4. The concentrations of compounds varied from 0 to 30 μM.

From the results of the Table 3 and Figure 4A–G, the quenching constant K_sv_ of these systems are decreased with increase in temperature indicating a static quenching mechanism of the protein fluorescence. Furthermore, values of k_q_ were calculated for the interaction of these compounds with BSA. k_q_ values were found to be of the order of 10^12^, which are 100 times greater than maximum scatter collision quenching constant value 2.0 × 10^10^ L mol^−1^ s^−1^. It indicates that compounds interact with BSA in the static quenching manner [20]. Equation (3) is used to calculate the binding constant (K_b_) and number of binding sites (n) [21]:

(3) $$\log\left(\frac{F_{0}-F}{F}\right)={\mathrm{logK}}_{b}+\mathrm{nlog}\left[Q\right],$$

where F and F_0_ are fluorescence intensities with and without quencher, respectively. K_b_ is binding constant, n is the number of binding sites per BSA molecule and [Q] is the concentration of compounds. Figure 5 shows the binding equilibrium plots for the fluorescence quenching of BSA by compounds **1**–**7** at 289, 297 K and 307 K. Values of K_b_ and n gained from Equation (3) are given in Table 3. The number of binding sites in all systems approximates to 1 indicating that only one binding site in protein is reactive to the compounds **1**–**7**. The binding constants K_b_ of 2–6 are decreased with increasing temperature, which was in accordance with the change of K_sv_. The K_b_ of compounds **1** and **7** are increased with the increasing temperature, which is denoted differently with respect to compounds **2**–**6**. 

### 2.4. Types of Interaction Forces between BSA and Compounds

The binding forces contributing to protein interactions with small molecular substrates often include van der Waals interactions, hydrophobic forces, electrostatic interactions, and hydrogen bonding. The signs and magnitudes of thermodynamic parameters, such as standard enthalpy change (∆H), standard entropy change (∆S) and free energy (∆G) of binding reaction provide evidence for binding mode between the small molecules and macromolecule. The thermodynamic parameters are evaluated using the the Van’t Hoff e equations [22]:

(4) $${\mathrm{LnK}}_{b}=-∆H/\mathrm{RT}+∆S/R,$$

(5) $$\Delta G=-{\mathrm{RTlnK}}_{b},$$

where K is the binding constant at the corresponding temperature, R is the gas constant, and T is absolute temperature. Due to the ΔH and ΔS signs of the system, the binding mechanism between a small molecule and protein can be determined. Accordingly, hydrophobic forces are dominant when ΔH > 0 and ΔS > 0, van der Waals interactions and hydrogen bonds contributed to binding when ΔH < 0 and ΔS < 0, and ΔH < 0 and ΔS > 0 are characteristics for electrostatic interactions [22,23]. The values of ∆H, ∆S and ∆G are listed in Table 3. The negative values of ΔG mean that the binding process is spontaneous. The negative ΔH and ΔS values indicate that hydrogen bonds and van der Waals forces played a major role in the interaction of the compounds **2**–**6** system. Both positive ∆S and ∆H in the case of compounds **1** and **7** suggest the presence of hydrophobic interactions.

### 2.5. Synchronous Fluorescence Spectroscopic Studies

**Figure 6:**
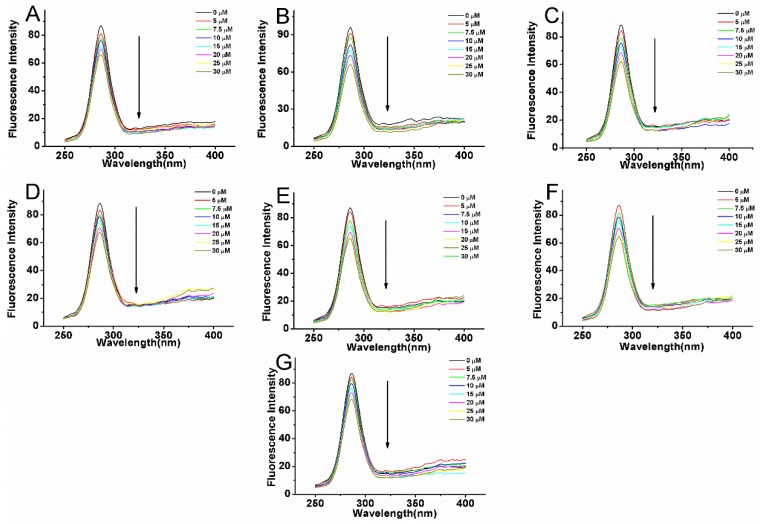
Synchronous fluorescence (Δλ = 60 nm) of BSA (5 μM) with variable concentrations of compounds **1** (**A**), **2** (**B**), **3** (**C**), **4** (**D**), **5** (**E**), **6** (**F**), **7**(**G**) at the excitation wavelength (280 nm) at room temperature in 0.05 mol L^−1^ Tris-HCl, pH 7.4. The concentrations of compounds varied from 0 to 30 μM.

**Figure 7:**
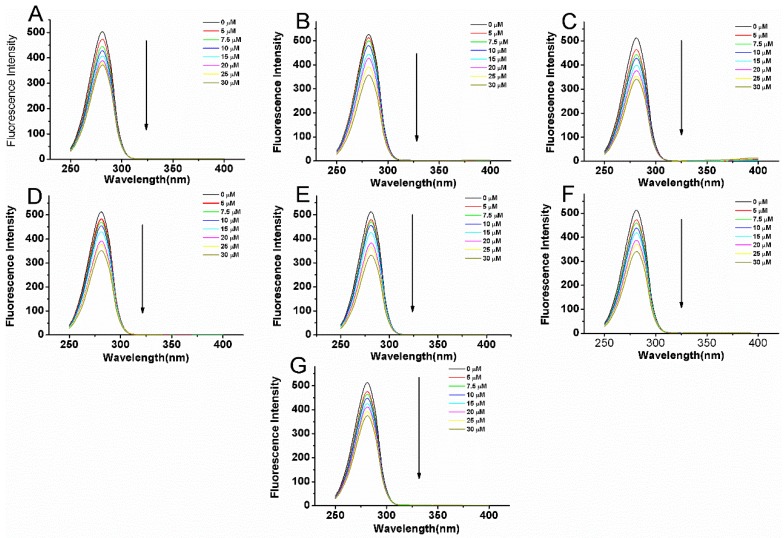
Synchronous fluorescence (Δλ = 15 nm) of of BSA (5 μM) with variable concentrations of compounds **1** (**A**), **2** (**B**), **3** (**C**), **4** (**D**), **5** (**E**), **6** (**F**), **7** (**G**) at the excitation wavelength (280 nm) at room temperature in 0.05 mol L^−1^ Tris-HCl, pH 7.4. The concentrations of compounds varied from 0 to 30 μM.

Synchronous fluorescence spectroscopy is a very useful method to study the microenvironment of amino acid residues by measuring the emission wavelength shift. The synchronous fluorescence of protein at Δλ = 15 nm and Δλ = 60 nm are characteristic of Tyr and Trp residues, respectively. The Δλ value represents the difference between excitation and emission wavelengths and is an important parameter [24]. The synchronous fluorescence spectra of BSA in the presence of different concentration of the compounds are shown in Figure 6 and Figure 7. It can be seen that the intensities of synchronous fluorescence gradually decreased upon addition of compounds, which is consistent with the steady state fluorescence results. At the same time, the maximum emission wavelength almost does not change when the Δλ was set at 15 nm. A slight red shift take place at the Δλ = 60 nm. However, no shift was observed in fluorescence quenching experiments, suggesting a similar microenvironment surrounding Trp residues upon ligands binding.

### 2.6. Circular Dichroism Studies

**Figure 8:**
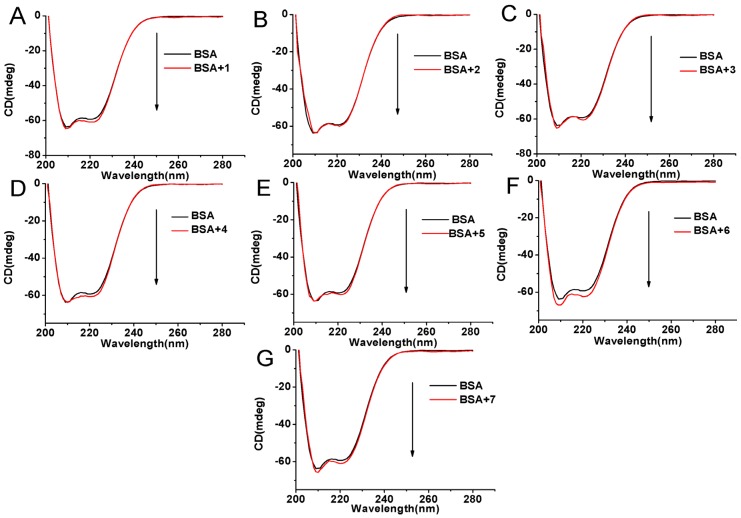
CD spectra of BSA (5 μM) in the presence of compounds **1** (**A**), **2** (**B**), **3** (**C**), **4** (**D**), **5** (**E**), **6** (**F**) and **7** (**G**) in 0.05 mol L^−1^ Tris-HCl, pH 7.4. The concentrations of the compounds were 30 μM.

In order to determine the effect of compounds binding on the secondary structure of BSA, a circular dichroism (CD) spectroscopic analysis was performed. As can be observed in Figure 8, the CD spectra of BSA exhibit two negative bands at 208 nm and 222 nm characteristic of α-helical structures in the protein to gain an understanding on the results of the CD spectra of BSA in the presence of compounds the α-helical content of BSA is evaluated from the following equations:

(6) $$\mathrm{MER}=\frac{\mathrm{CD}\left(\mathrm{mdeg}\right)}{10\cdot n\cdot l\cdot C_{p}},$$

(7) $$\alpha -\mathrm{helix}\left(\%\right)=\frac{-{\mathrm{MER}}_{208}-4000}{33000-4000}\times 100,$$

where MRE_208_ is mean residue ellipticity observed at 208 nm., C_p_ is the molar concentration of the protein, *n* is the number of amino acid residues, and l is the path length of the cell. 4000 and 33,000 are the MRE values of a β-form with random coil conformation and a pure α-helix at 208 nm, respectively. 

A molar ratio of 1:6 for BSA: compounds was used for the CD measurements. From the above equations, the α-helix contents of BSA for the compound-BSA complexes are 60.12% for **1**, 59.01% for **2**, 60. 08% for **3**, 59.28% for **4**, 59.59% for **5**, 64.17% for **6**, and 60.49% for **7**, which are slightly changed compared with the native BSA value (58.61%). It can be seen from the data that the binding of compounds with BSA causes slight conformational change. These results are in agreement with those obtained from synchronous fluorescence spectra.

### 2.7. Energy Transfer from BSA to Compounds

**Figure 9:**
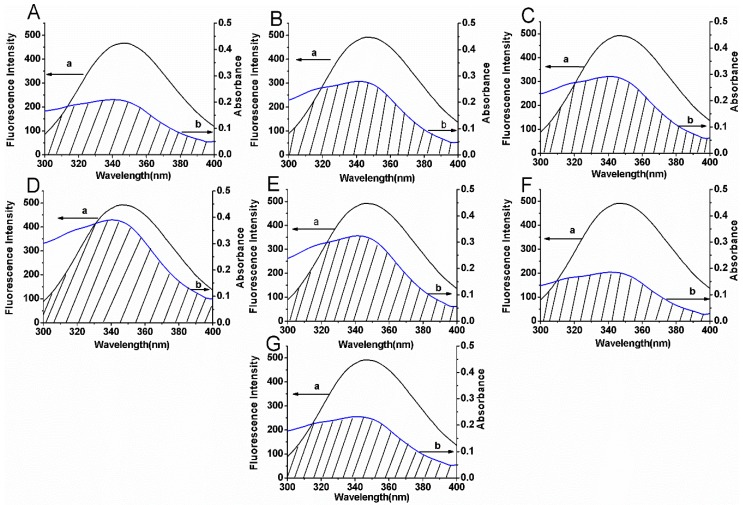
Spectral overlaps between the fluorescence spectra (a) of BSA and the absorption spectra (b) of compounds **1** (**A**), **2** (**B**), **3** (**C**), **4** (**D**), **5** (**E**), **6** (**F**) and **7** (**G**) in 0.05 mol L^−1^ Tris-HCl, pH 7.4.The concentrations of BSA and compounds are 5 μM and 30 μM, respectively.

Förster’s non-radiative energy transfer theory is widely used to estimate the spatial distances between a biomolecule and a small molecule. The energy transfer efficiency (E) from the donor (BSA) to the acceptor (the compounds) can be determined by the following equation [25,26]:

(8) $$E=1-\frac{F}{F0}=R_{0}^{6}/\left(r^{6}+R_{0}^{6}\right),$$

where E is the energy transfer efficiency, F and F_0_ are the fluorescence intensities of BSA in the presence and absence of compounds, r is the distance between acceptor and donor and R_0_ is the critical distance when the transfer efficiency is 50%. The R_0_ can be calculated by Equation (10):

(9) $$R_{0}^{6}=8.8\times 10^{-25}k^{2}N^{-4}\Phi J,$$

where k^2^ is the spatial orientation factor of the dipole with k^2^ = 2/3, N is the refractive index of the medium and its value is 1.366, Φ is the fluorescence quantum yield of the donor and Φ = 0.118. J is the overlap integral of the fluorescence emission spectrum of the donor and the absorption spectrum of the acceptor [25]. J is given by Equation (7):

(10) $$J=\frac{\sum^{\text{​}}F\left(\lambda \right)\varepsilon \left(\lambda \right)\lambda^{4}\Delta \lambda }{\sum^{\text{​}}F\left(\lambda \right)\Delta \lambda },$$

where F(λ) is the fluorescence intensity of the fluorescent donor at wavelength, and ε(λ) is the molar absorption coefficient of the acceptor at wavelength. The overlap of the absorption spectra of compounds and the fluorescence emission spectra of BSA are shown in Figure 9 A–G. The calculated R_0_ and r binding distances are showed in Table 4. A transfer of energy could take place through direct electrodynamic interaction between the primarily excited molecule and its neighbors, when the distance between the donor and the acceptor is approach in the range of 2–8 nm. As the distances between BSA and compounds are **2**–**8** nm scale and 0.5R_0_ < r < 1.5R_0_, suggesting that the energy transfer from BSA to compounds occurs with high probability. The calculated r-value was larger than that of R_0_, further indicating that the fluorescence quenching of BSA induced by compounds **1**–**7** is the result of a static quenching mechanism [27,28].

### 2.8. The determination of Binding Sites of Compound ***6*** on BSA

The BSA has been previously reported to have two major specific drug-binding sites, which are defined as site I and site II, respectively. Binding site location for compound **6** was analyzed through competitive binding assay and molecular docking, since this compound has the strongest activity. Warfarin as the marker for site I and ibuprofen for site II was used in this experiment, respectively. The concentration of the markers and BSA were both 5 µM, while the concentration of the compound was 0–30 µM. Fluorescence emission spectra of the mixed solutions of BSA and site markers following a concentration increment of compound **6** were recorded and the results were presented in Table 5. It can be observed that the K_b_ and log K_b_ values of the compound with BSA decreased markedly in the presence of warfarin and ibuprofen, indicating that there are competitive interactions between compound **6** and the two site maker with BSA. Therefore, it can be deduced that the compound **6** may interact with BSA at both site I and site II. Nevertheless, the value of K_b_ is much lower in the presence of warfarin, suggesting that site I is the main binding site. 

**Figure 10:**
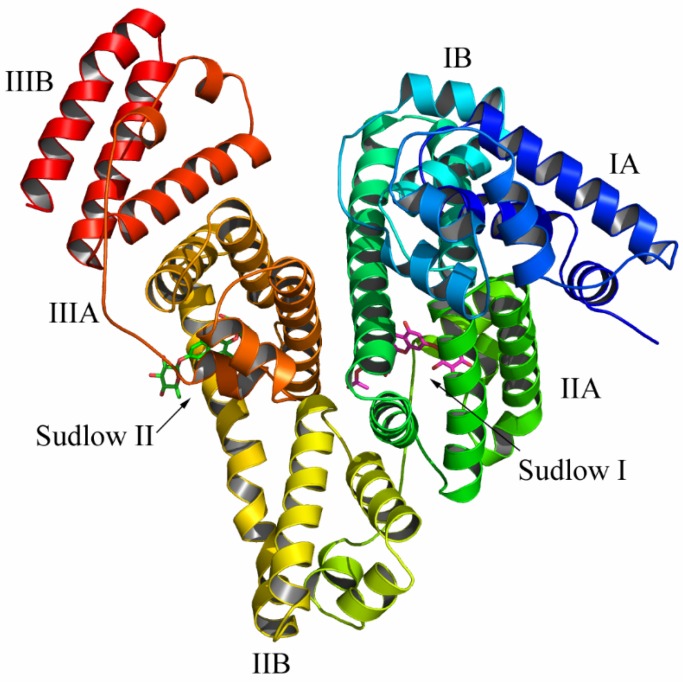
Compound **6** was docked into the Site I (IIA) and Site II (IIIA) binding pockets of the BSA.

**Figure 11:**
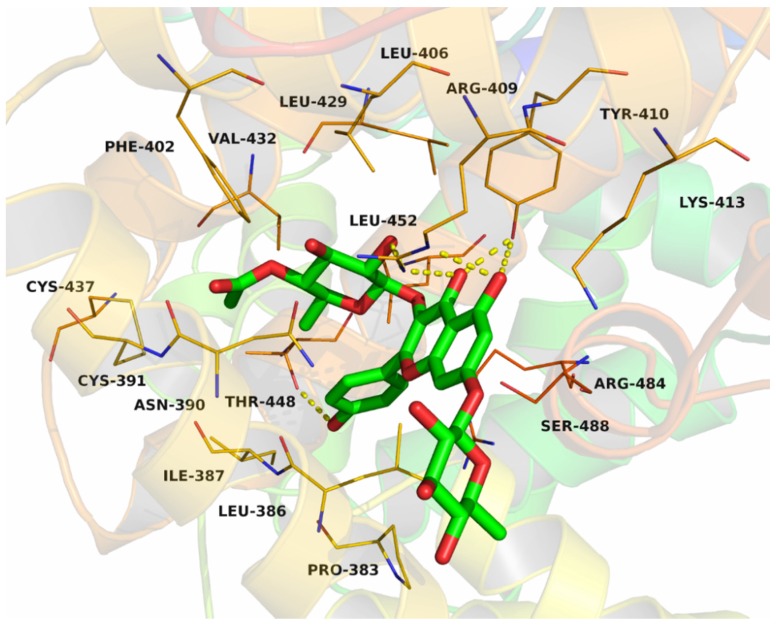
Compound **6** was docked into the Site II (IIIA) binding pocket of the BSA.

Compound **6** was docked into the Site II (IIIA site) and Site I (IIA site) binding sites of the BSA, respectively (Figure 10). The detailed binding mode of compound **6** to the Site II binding site in subdomain IIIA of the BSA was shown in Figure 11. The acetyl group of compound **6** fit at the bottom of the BSA pocket and made a high density of van der Waals contacts, whereas the rhamnopyranosyl group of compound **6** was positioned at the entrance of the pocket and makes only a few contacts. Detailed analysis showed that the acetyl group of compound **6** stretched into the hydrophobic pocket that consisted of Cys-391, Phe-402, Val-432 and Cys-437, while the 4-hydroxylphenyl group of compound **6** was located at another hydrophobic pocket, surrounded by the residues Pro-383, Leu-386, Ile-387 and Leu-452, forming a stable hydrophobic binding. In addition, cation-π interactions were observed between the kaempferol scaffold of compound **6** and the residues Arg-409, Lys-413 and Arg-484. Importantly, six hydrogen bond interactions were observed between the compound **6** and the residues Thr-448, Arg-409 and Tyr-410 of the BSA.

**Figure 12:**
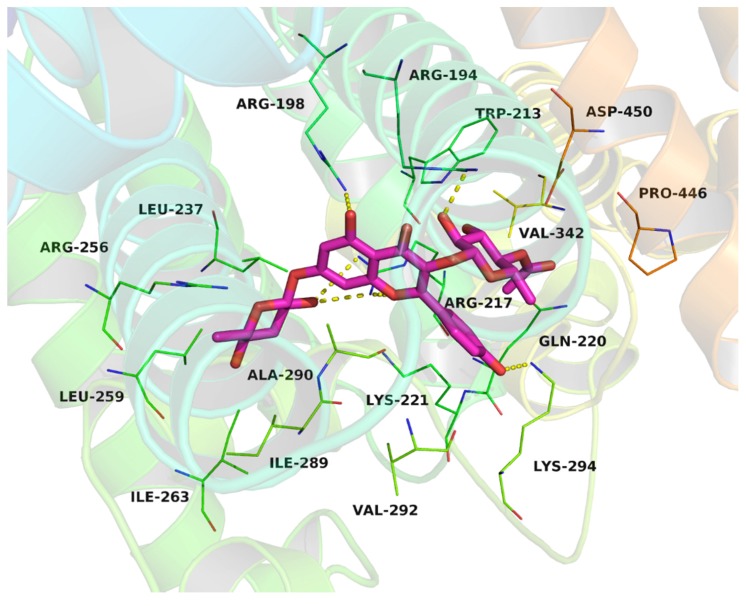
Compound **6** was docked into the Site I (IIA) binding pocket of the BSA.

To gain further insight into the binding interaction between compound **6** and the BSA in the molecular level, compound **6** was docked to the Site I of the BSA (Figure 12). The rhamnopyranosyl group of compound **6** fit at the bottom of the BSA pocket and made a high density of van der Waals contacts, whereas the other two sides of compound 6 were positioned near the entrance of the pocket and makes only a few contacts. Detailed analysis showed that the acetyl group of compound **6** stretched into the hydrophobic pocket that consisted of Trp-213, Val-342 and Pro-446, while the kaempferol scaffold of compound **6** was located at the hydrophobic pocket, surrounded by the residues Ile-289, Ala-290 and Val-292, forming a stable hydrophobic binding. Moreover, cation-π interactions were observed between the kaempferol scaffold of compound **6** and the residues Arg-194, Arg-198, Arg-217, Lys-221 and Lys-294. Importantly, six hydrogen bond interactions were observed between the compound **6** and the residues Arg-194, Arg-198, Arg-217, and Lys-294. All of these interactions helped compound **6** to anchor in the binding site of BSA. Molecular docking analysis suggests a better binding interaction for compound **6** with site I, and this is consistent with the results of competitive binding assay. 

In summary, the above molecular simulations give us a rational explanation of the interactions between compound **6** and the BSA, which will provide a good structural basis to explain the fluorescence quenching of BSA emission in the presence of compound **6**. 

The present study provided the detailed information about the binding characteristics and of compounds **1**–**7** to BSA, which provides useful data for future pharmacological studies.

## 3. Materials and Methods

### 3.1. Chemicals and Instrumentation

Fatty acid-free BSA was obtained from Shanghai Shenhang Biotechnology Co., Ltd. (Shanghai, China). The stock solution of compounds **1**–**7** was prepared in distilled water, respectively. All of the above solutions were kept in the dark at 0–4 °C. Tris–HCl buffer solution (0.05 mol L^−1^, pH 7.4) containing 0.1 mol L^−1^ NaCl were used. All reagents were of analytical reagent grade without further purification and double distilled water was used throughout the experiment.

IR spectra were recorded on a Thermo-Nicolet 670 spectrophotometer (Madison, Wi. USA,) using KBr disks. ^1^H and ^13^C-NMR spectra were recorded on a Bruker Advance 600 spectrometer (Fällanden, Switzerland) at 600 (^1^H) and 150 (^13^C) MHz, respectively. High Resolution Electrospray ionization Mass (HRESIMS) spectra were measured on an LTQ-Orbitrap XL (Thermo Electron., San Jose, CA, USA). Fluorescence measurements were carried out on Cary Eclipse Fluorescence spectrophotometer (Varian Australia Pty Ltd., Mulgrave, Australia), using a 1 cm quartz cell. The circular dichroism (CD) spectra were recorded using Chirascan CD spectrophotometer (Applied Photophysics Ltd., Leatherhead, UK). Semi preparative HPLC was performed on an Agilent 1200 liquid chromatograph (Germany) with a ZORBAX SB-C18 column (Agilent, CA, USA) (9.4 mm × 250 mm, 5 μm).

### 3.2. Plant Material

The roots of *W. unigemmata* were collected in Linyi, Shandong Province, P.R. China, in July 2015 and identified by Dr. Tao Shen, School of Pharmaceutical Sciences, Shandong University. A voucher specimen (No. 201507GZ01) has been deposited at the Laboratory of Department of Natural Products Chemistry, School of Pharmaceutical Sciences, Shandong University, China.

### 3.3. Extraction and Isolation

Powder of the dry rhizomes (2.0 kg) was extracted three times (72 h each) with EtOH-H_2_O (90:10) at room temperature. After evaporation of the solvent in vacuo, the concentrate was suspended into H_2_O and partitioned successively with petroleum ether (60–90 °C) (2000 mL × 3), EtOAc (2000 mL × 3) and *n*-BuOH (2000 mL × 3). The n-BuOH layer was evaporated in vacuo to yield *n*-BuOH fraction (15.0 g). The *n*-BuOH fraction was subjected to silica gel chromatographic column (CC) using a gradient of CH_2_Cl_2_–MeOH (95:5–50:50) to give 7 sub-fractions (Fr.1–Fr.7). Sub-fraction Fr.2 was purified by semi-HPLC using acetonitrile-H_2_O (40:60) at a flow rate of 2 mL/min to afford compound **1** (t_R_ 9.5 min, 12 mg). Sub-fraction Fr.3 was separated by HPLC using acetonitrile-H_2_O (28:72) at a flow rate of 2 mL/min to obtain compound **2** (t_R_15.6 min, 13 mg), and 3 (t_R_ 17.0 min, 8 mg). Sub-fraction Fr.4 was conducted by HPLC using acetonitrile-H_2_O (22:78) at a flow rate of 2 mL/min to produce compound **4** (t_R_ 12.4 min, 8 mg), 5 (t_R_ 14.2 min, 8.5 mg), and 6 (t_R_ 16.8 min, 10 mg). Sub-fraction Fr.5 was subjected to HPLC using acetonitrile-H_2_O (22:78) at a flow rate of 2 mL/min to afford compound **7** (t_R_ 5.7 min, 9 mg).

Compound **1**: Yellow amorphous powder; UV (MeOH) λ_max_ (logε) 265 (3.42), 340 (2.05) nm; IR (KBr) ν_max_ 3382, 2935, 1723, 1596, 1175 cm^−1^; ^1^H and ^13^C NMR see Table 1; HR-ESI-MS *m*/*z* 705.2027 [M + H]^+^ (calcd for C33H37O17, 705.2025).

### 3.4. Sugar Identification

l-Rhamnose (72 mg) and l-cysteine methyl ester hydrochloride (90 mg) were dissolved in pyridine (3 mL) and stirred at 60 °C for 1.5 h, and then *o*-tolyl isothiocyanate (360 µL) was added to the mixture and heated at 60 °C for 1.5 h. Separation by HPLC using a C-18 column eluted with H_2_O (containing 0.2% TFA)-CH3CN (65:35) gave the derivative S-1.

Compound **1** was hydrolyzed with 1% HCl and extracted with CH_2_Cl_2_. The aqueous layer was passed through a Sephadex LH-20 column (Agilent, CA., USA) and the eluate was concentrated. The residue was dissolved in pyridine (0.5 mL) and stirred with L-cysteine methyl ester (10 mg) for 1.5 h at 60 °C, and then *o*-tolyl isothiocyanate (40 µL) was added to the mixture and heated at 60 °C for 1.5 h. The reaction mixture was analyzed by HPLC and detected by UV absorbance at 250 nm. Analytical HPLC was performed on a Phenomenex C18 column (4.6 × 250 mm) (Torrance, CA, USA) at 30 °C using a gradient of CH_3_CN: 0.15% TFA in H_2_O: 0–20 min (30:70), 20–30 min (30:70 to 70:30), 30–60 min (70:30) as the mobile phase. Peaks were detected with an Agilent DAD detector. l-rhamnose was identified as the sugar moieties of 1 as they had the same retention times with S-1 (t_R_ 16.1 min) [12].

### 3.5. MTT Cytotoxicity Assay

The human leukemia cell line K562, and its multidrug-resistant counterpart K562/A02 were obtained from the Department of Pharmacology, the Institute of Hematology of Chinese Academy of Medical Sciences (Tianjin, China). K562/A02 cells were maintained in a complete RPMI-1640 medium containing 1 µg/mL doxorubicin at 37 °C in a humidified atmosphere of 5% CO_2_. The cells were cultured for two weeks in drug-free medium prior to their use in the experiments.

Cells were harvested and seeded into 96-well plates at 2 × 10^4^ cells/well. For cytotoxicity experiments, different concentrations (10, 20, 40, 80, and 160 μM) of compounds **1**–**7** were added into designated wells, and for MDR reversal experiments, different concentrations (0.1, 0.2, 0.5, 1, 5, 10, 20, and 40 μM) of doxorubicin (DOX) were added into designated wells with or without compounds **1**–**7** (10 μM). After 48 h, 3-(4,5-dimethylthiazol-2-yl)-2,5-diphenyltetrazolium bromide (MTT) solution was added to each well, and the plate was further incubated for 4 h. The medium was discarded, and 200 μL of dimethyl sulphoxide (DMSO) was added into each well to dissolve the formazan crystal. The absorbance in individual wells was determined at 570 nm with a Micro-Reader (BioTek instruments, Inc., Winooski, VA., USA). IC_50_ values (concentration resulting in 50% inhibition of cell growth) for compounds **1**–**7** and doxorubicin were calculated from plotted results using untreated cells as 100%. The reversal fold (RF) values, as potency of reversal, were obtained from fitting the data to RF = IC_50_ of cytotoxic DOX alone/IC_50_ of cytotoxic DOX in the presence of the tested compounds [10].

### 3.6. BSA Binding Experiment

Fluorescence measurements were performed using a 1 cm quartz cell. The widths of excitation and emission slit were set at 5.0 nm. The excitation wavelengths were set at 280 nm, and the emission wavelength was recorded between 290 and 450 nm. The fluorescence intensities were corrected for inner filter and dilution effects before analysis of the binding and quenching data. The concentration of BSA during the experiment was 5 μM. Molar ratios of drug to protein used were 0, 1, 1.5, 2, 3, 4, 5 and 6.

Synchronous fluorescence spectra of all BSA solutions in the absence and presence of compounds were obtained were recorded on Varian Cary Eclipse with 1 cm quartz cell at room temperature. The scanning intervals of ∆λ (∆λ = λ_em_ − λ_ex_) were set at 15 and 60 nm, which were used to characterize the properties of the tyrosine and tryptophan residues, respectively.

CD Spectra of free BSA (5 μM) and compounds (30 μM) were obtained by a Chirascan CD spectrophotometer (Applied Photophysics Ltd., Leatherhead, UK) at room temperature. A quartz cell with a path length of 1 mm was used to do measurements in the wavelength range of 200–400 nm. Scan speed to obtain spectra was maintained at 164 nm/min with an interval of 1 nm. 

Absorption measurements were carried out at room temperature on Shimadzu UV-2500 UV-VIS Spectrophotometer (Suzhou, Jiangsu, China) using a 10 mm cell. Wavelength was recorded between 300 and 400 nm.

### 3.7. Molecule Modeling

Crystal structure of BSA (PDB ID: 4JK4) was obtained from Protein Data Bank [29]. The 2D structure of the compound **6** was drawn by ChemBioDraw Ultra 12.0 (2010, CambridgeSoft, Cambridge, MA., USA) and converted to 3D structure by ChemBio3D Ultra 12.0 software (CambridgeSoft, Cambridge, MA., USA). The binding interactions of compound **6** with BSA were simulated by a docking method implemented in AutoDock Vina (1.1.2, The Scripps Research Institute, La Jolla, CA., USA) along with MGLTools (1.5.6, The Scripps Research InstituteManufacturer, La Jolla, CA., USA) [30,31,32]. A ligand was prepared for docking by merging non-polar hydrogen atoms and defining rotatable bonds. The search grid of the BSA IIIA site (Site II) was identified as center_x: 100.77, center_y: 28.293, and center_z: 36.82 with dimensions size_x: 15, size_y: 15, and size_z: 15, and the IIA site (Site I) was identified as center_x: 95.873, center_y: 20.925, and center_z: 17.631 with dimensions size_x: 15, size_y: 15, and size_z: 15. In order to increase the docking accuracy, the value of exhaustiveness was set to 20. For Vina docking, the default parameters were used if it was not mentioned. The best-scoring pose as judged by the Vina docking score was chosen and visually analyzed using PyMoL software (1.3r1, DeLano Scientific LLC, South San Francisco, USA) [6].

## 4. Conclusions

In this work, seven compounds were isolated from the roots of Woodwardia unigemmata. The structures of the compounds were elucidated by spectroscopic analysis, as well as by comparison with literature data. Compound **6** showed comparable MDR reversing effect to verapamil. The interactions between complexes **1**–**7** and BSA were investigated employing different spectroscopic and molecular docking techniques. The experimental results indicated that these compounds bind to BSA by static quenching mechanisms. The negative ΔH and ΔS values indicated that van der Waals interactions and hydrogen bonds contributed in the binding of compounds **2**–**6** to BSA. On the other hand, for compounds **1** and **7**, the hydrophobic interactions play a major role on BSA binding. There are two binding sites of compound 6 on BSA and the site I is the main one through the molecular docking and competitive binding assay. Meanwhile, there is a slight change in the secondary structure of BSA after the binding of compound **6**. 

The present study provided the detail information about the binding characteristics and conformation of compounds **1**–**7** on BSA, which provide useful data for future pharmacological studies.

## Figures and Tables

**Table 1:** ^1^H-NMR (600 MHz) and ^13^C-NMR (150 MHz) data of compound **1** in CD_3_OD.

Position	δ_H_ (*J* in Hz)	δ_C_	HMBC (H → C)
2		158.5	
3		134.0	
4		178.1	
5		161.6	
6	6.52 d (1.8)	99.2	C-5, C-7, C-8
7		162.2	
8	6.79 d (1.8)	94.2	C-7, C-10, C-6
9		156.7	
10		106.2	
1′		120.8	
2′	7.82 d (8.4)	130.6	C-1′, C-3′/5′, C-4′
3′	7.00 d (8.4)	115.3	C-1′, C-2′/6′, C-4′
4′		160.6	
5′	7.00 d (8.4)	115.3	C-1′, C-2′/6′, C-4′
6′	7.82 d (8.4)	130.6	C-1′, C-3′/5′, C-4′
1′′	5.64 d (1.2)	100.5	C-3, C-2′′
2′′	4.36 m	67.7	C-1′′, C-3′′
3′′	5.18 dd (10.2 3.0)	71.3	C-4′′, 3′′-COCH_3_
4′′	5.05 t (10.2)	70.3	C-5′′, 4′′-COCH_3_
5′′	3.78 m	68.3	C-4′′, C-6′′
6′′	0.84 d(6.0)	16.1	C-4′′, C-5′′
1′′′	5.58 m	98.5	C-7, C-2′′
2′′′	4.24 m	73.3	C-1′′′, C-3′′′,
3′′′	4.06 dd (9.6 3.0)	70.4	C-2′′′, C-4′′′
4′′′	4.78 t (9.6)	73.3	C-3′′′, 4′′′-COCH_3_
5′′′	3.33 m	69.9	C-4′′′, C-6′′′
6′′′	1.17 d (6.0)	16.7	C-5′′′
3′′-COCH_3_		170.7	
3′′-COCH_3_	2.10 s	19.4	3′′-COCH_3_
4′′-COCH_3_		170.3	
4′′-COCH_3_	2.00 s	19.2	4′′-COCH_3_
4′′′-COCH_3_		170.9	
4′′′-COCH_3_	1.96 s	19.5	4′′′-COCH_3_

**Table 2:** Effects of compound **1**–**7** (10 μM) on doxorubicin (DOX) cytotoxicity in K562 cells and K562/A02cells. Half maximal inhibitory concentration(IC _50_) values (μM) for DOX were calculated and the reversal folds (RF) were evaluated. Data were expressed as means ± SD of five independent experiments.

Compounds	IC_50_ (K562)	IC_50_ (K562/A02)	RF (K562/A02)
DOX	0.66 ± 0.12	19.2 ± 0.31	
DOX + Verapamil	0.58 ± 0.23	2.12 ± 0.19	9.06
DOX + 1	0.67 ± 0.11	5.18 ± 0.17	3.71
DOX + 2	0.62 ± 0.08	4.14 ± 0.33	4.64
DOX + 3	0.69 ± 0.10	3.74 ± 0.12	5.13
DOX + 4	0.70 ± 0.24	3.04 ± 0.33	4.75
DOX + 5	0.65 ± 0.16	5.14 ± 0.12	3.74
DOX + 6	0.68 ± 0.12	2.31 ± 0.10	8.31
DOX + 7	0.60 ± 0.22	3.93 ± 0.26	5.94

**Table 3:** Binding and thermodynamic parameters for the interaction between compounds **1**–**7** and BSA at different temperatures, pH = 7.4.

NO	T (K)	K_sv_ (×10^4^ L·mol^−1^)	R^2^	K_q_ (×10^12^ L·mol^−1^·s^−1^)	Log K_b_	K_b_ (×10^5^ L·mol^−1^)	n	R^2^	ΔG (KJ·mol^−1^)	ΔH (KJ·mol^−1^)	ΔS (J·mol^−1^·K^−1^)
**1**	289	1.6	0.994	1.6	4.472	0.3	1.06	0.994			
	297	1.49	0.991	1.49	4.616	0.41	1.1	0.992	−26.25	67.1	316.68
	307	1.3	0.995	1.3	5.082	1.21	1.22	0.996			
**2**	289	2.76	0.996	2.76	5.332	2.15	1.1	0.986			
	297	2.24	0.995	2.24	4.757	0.57	1.09	0.997	−27.05	−84.47	−191.14
	307	1.79	0.993	1.79	4.589	0.39	1.06	0.991			
**3**	289	2.77	0.993	2.77	4.701	0.5	1.06	0.995			
	297	2.26	0.99	2.26	4.675	0.47	1.07	0.997	−26.58	−29.55	−11.55
	307	1.99	0.992	1.99	4.43	0.27	1.03	0.991			
**4**	289	2.5	0.993	2.5	5.265	1.84	1.2	0.985			
	297	2.14	0.998	2.14	5.027	1.06	1.16	0.996	−28.58	−89.64	−208.18
	307	1.87	0.992	1.87	4.453	0.28	1.04	0.998			
**5**	289	2.91	0.995	2.91	5.277	1.89	1.19	0.998			
	297	2.4	0.996	2.4	4.984	0.96	1.14	0/995	−28.34	−54.79	−88.71
	307	2.14	0.996	2.14	4.79	0.62	1.11	0.993			
**6**	289	2.29	0.996	2.29	4.894	0.78	1.08	0.997			
	297	2.11	0.993	2.11	4.662	0.46	1.08	0.996	−26.69	−38	−38.1
	307	1.75	0.995	1.75	4.558	0.36	1.07	0.996			
**7**	289	2.74	0.998	2.74	3.819	0.07	0.9	0.997			
	297	1.53	0.996	1.53	4.327	0.21	1.03	0.998	−24.6	90.73	387.43
	307	1.4	0.998	1.4	4.624	0.42	1.11	0.998			

**Table 4:** Energy transfer parameters of the different compounds—BSA complexes.

Compounds	J (cm^3^ L M^−1^)	R_0_ (nm)	r (nm)	E (%)
1	7.70 × 10^−15^	2.35	2.72	29.30
2	9.90 × 10^−15^	2.45	2.62	39.97
3	1.05 × 10^−14^	2.47	2.62	41.25
4	1.44 × 10^−14^	2.60	2.79	39.69
5	1.16 × 10^−14^	2.51	2.69	39.98
6	6.47 × 10^−15^	2.28	2.42	41.23
7	8.50 × 10^−15^	2.39	2.64	35.20

**Table 5:** Binding constants of BSA with compound **6** in the presence of site markers in Tris-HCl buffer (pH = 7.4) at room temperature.

System	LogK_b_	K_b_ (×10^5^ L mol^−1^)	R^2^
BSA + compound **6**	4.662	0.459	0.993
BSA + compound **6** + ibuprofen	4.324	0.211	0.989
BSA + compound **6** + warfarin	3.227	0.017	0.993
